# [Retracted] Genistein inhibits lung cancer cell stem-like characteristics by modulating MnSOD and FoxM1 expression

**DOI:** 10.3892/ol.2025.15097

**Published:** 2025-05-19

**Authors:** Zhimin Fu, Xiaocheng Cao, Lihua Liu, Xiaozheng Cao, Yinghong Cui, Xiang Li, Meifang Quan, Kaiqun Ren, A Chen, Chang Xu, Yebei Qiu, Xiangding Chen, Zheng Wang, Jianguo Cao

Oncol Lett 20: 2506–2515, 2020; DOI: 10.3892/ol.2020.11802

Following the publication of this paper, it was drawn to the Editor's attention by a concerned reader that western blot data featured in Figs. 4C, 5A and 6C were strikingly similar to data that had already appeared in a pair of previously published articles written by different authors at different research institutes; in addition, three pairs of data panels showing the results of cell migration and invasion assay experiments in Figs. 1G, 2F, 3F and 5F were found to be overlapping, where data intended to show the results from differently performed experiments had apparently been derived from the same original source(s). Owing to an overall lack of confidence in the presented data, and due to the fact that the abovementioned data in the above article had already been published prior to its submission to *Oncology Letters*, the Editor has decided that this paper should be retracted from the Journal. The authors were asked for an explanation to account for these concerns, but the Editorial Office did not receive a reply. The Editor apologizes to the readership for any inconvenience caused.

